# Approaches to Foster Transfer of Formal Principles: Which Route to Take?

**DOI:** 10.1371/journal.pone.0148787

**Published:** 2016-02-12

**Authors:** Lennart Schalk, Henrik Saalbach, Elsbeth Stern

**Affiliations:** 1 ETH Zurich, Zurich, Switzerland; 2 Leipzig University, Leipzig, Germany; University of Leuven, BELGIUM

## Abstract

Enabling learners to transfer knowledge about formal principles to new problems is a major aim of science and mathematics education, which, however, is notoriously difficult to reach. Previous research advocates different approaches of how to introduce principles to foster the transfer of knowledge about formal principles. One approach suggests teaching a generic formalism of the principles. Another approach suggests presenting (at least) two concrete cases instantiating the principle. A third approach suggests presenting a generic formalism accompanied by a case. As yet, though, empirical results regarding the transfer potential of these approaches are mixed and difficult to integrate as the three approaches have rarely been tested competitively. Furthermore, the approaches have been evaluated in relation to different control conditions, and they have been assessed using varying transfer measures. In the present experiment, we introduced undergraduates to the formal principles of propositional logic with the aim to systematically compare the transfer potential of the different approaches in relation to each other and to a common control condition by using various learning and transfer tasks. Results indicate that all approaches supported successful learning and transfer of the principles, but also caused systematic differences in the magnitude of transfer. Results indicate that the combination of a generic formalism with a case was surprisingly unsuccessful while learners who compared two cases outperformed the control condition. We discuss how the simultaneous assessment of the different approaches allows to more precisely capture the underlying learning mechanisms and to advance theory on how these mechanisms contribute to transfer performance.

## Introduction

Many academic domains, foremost science and mathematics, are based on abstract, formal principles such as Newtonian laws, complex systems principles, or propositional logic. These kinds of principles are highly generalizable; that is, they can be used to understand and deal with a multitude of superficially dissimilar situations and problems. Essentially, principles are generalizable as they capture the interdependencies and relations of situations rather than their superficial characteristics (e.g., [1]). A major goal of education is to help learners acquire relational knowledge about formal principles (i.e., schemata, structures) which would allow for flexible generalization [2]. Put differently, education aims at enabling learners to use and apply their knowledge about formal principles to a wide range of novel problems and situations.

In this sense, transfer of relational knowledge about principles can be understood as the ability to productively use one’s knowledge [3] to solve novel tasks which *look* different, but are actually analogs and thus share the relational structures of the tasks which have been studied initially [4]. There is, however, overwhelming research (for an overview see [5]) showing that spontaneous analogical transfer is far from natural even if tasks differ only slightly in superficial features, and difficult to achieve when the context in which the tasks are embedded is dissimilar. This lack of transfer has been attributed to learners’ difficulties in identifying the relational structure of a source problem presented as an initial learning opportunity or to learners’ failure to retrieve the learned knowledge because they are misled by irrelevant surface features of a target problem (see [6–8]). In the present study, different approaches to represent formal principles in learning materials were systematically compared with the aim to more precisely render whether and to what extent and how they support the ability to transfer relational knowledge about formal principles across a variety of tasks.

### Three different approaches to foster transfer of knowledge about formal principles

Cognitive and educational psychology have developed various approaches to promote flexible transfer of relational knowledge. We focus on three approaches that are well-described by previous research and are common in educational settings. More specifically, we compared the transfer potential that results from (1) learning a generic formalism of a principle only, (2) learning by comparing two cases that are presented simultaneously, and (3) learning a combination of a generic formalism and a single case. We refer to these approaches as (1) GENERIC, (2) 2CASES_SIM, and (3) CASE&GENERIC.

The GENERIC-approach promotes to introduce a novel principle with a a representation that has two properties: (1) it formalizes or symbolizes a principle by using variables and (2) it lacks direct reference to concrete objects or contexts; that is, it reduces the principle to its formal structure [9]. For example, Newton’s second law can be represented generically as “F = m * a” or a conjunction of two sentences can be represented generically as “p ∧ q” in propositional logic. Note, that according to our definition also graphical or pictorial representations can be represented generically. Thus, a two-dimensional coordinate systems in which the axes are labelled with x and y or a pictorial representation of an electric circuit in which a lamp, for instance, is represented as by a specific symbol are examples of generic, idealized representations. Starting with idealized, generic formalisms of principles is, for example, common in mathematics textbooks [10]. Several researchers reported advantages in transfer performance for learners who studied generic formalisms or idealized representations compared to learners who studied the formalism instantiated by concrete objects or embedded in concrete contexts (see [11–13]).

Researchers have hypothesized that “the power of symbols derives from the fact that they are virtually never identical to their referents” ([14], p. 33). Thus, the knowledge representation encoded from studying a generic representation of scientific principles is inferred to provide referential flexibility comparable to the principle itself. Indeed, proponents of this approach “argue that learning and transfer can be facilitated when knowledge is expressed in an abstract, generic form” ([15], p. 508). However, this approach might make it difficult for learners to connect the novel knowledge (i.e., the idealized symbolic notation) to their knowledge about specific situations or problems for which the principle might or might not be relevant [9]. Furthermore, it is important to note that generic representations also have specific (perceptual) properties [16]. That is, even though a formula idealizes the description of a principle from concrete situations and contexts, a formula *is* written in specific font or, simply, a formula *is* represented by variables and symbols.

The 2CASES_SIM-approach suggests to presenting learners with two (or more) concrete instantiations of the to-be-learned relational principle and to prompt the comparison of the two instantiations (e.g., [17–19]). A concrete instantiation grounds a principle in a specific context or situation [9]. We use the term *case* to refer to grounded representations of a principle. For example, learners have to simultaneously process two situations in which Newton’s second law is illustrated or they are presented with two cases in which a logical conjunction is used (e.g., simply two sentences connected by a conjunctive “and” instead of presenting the generic formalism “p ∧ q”) and are prompted to list similarities and differences of the two cases. We also consider a two-dimensional coordinate system in which the axes are labelled with content-related concepts (e.g., distance and time), or a pictorial representation of an electric circuit in which a picture of a concrete lamp is shown as cases. If learners actively compare cases transfer performance increases in comparison to studying the same cases sequentially (for a recent meta-analysis see [20]).

Researchers have hypothesized that presenting two or more cases simultaneously triggers a structural alignment and mapping process [19, 21]. This process helps learners to identify the common relational structure of the cases and thus, supports encoding of an abstracted schema [6]. Consequently, proponents of this approach argue that “the comparison process can be an efficient means of abstracting principles for their later application” ([22], p. 586). However, using cases to introduce novel principles entails the risk of conveying extra information that goes above and beyond the information that is captured by an abstract, formal principle. Therefore, transfer can be hampered by superficial irrelevant dissimilarities between learning and transfer tasks (e.g., [23]).

This problem could be overcome by the CASE&GENERIC-approach in which a single case is presented together with a generic formalism. This third approach thus combines the former two approaches in the most basic form. Recent research investigating this approach (e.g., [24, 25]) has mostly examined differences between learning with a combination of generic and concrete representations of a principle to learning one of the single representations within multimedia environments.

Analogous to the learning mechanisms assumed to drive the effectiveness of the 2CASES_SIM approach, researchers have hypothesized that CASE&GENERIC learners actively abstract and construct a schema by aligning the different representations into a coherent structure (sometimes referred to as coherence formation, see [26]). In addition to this “constructing” function, different representations can contain complementary information. For example, the concrete information provided by the case might activate prior knowledge; a generic representation might highlight a principle’s essential components and relations. However, one representation can also constrain the interpretation of the other (i.e., the so-called constraining function, see [27]). It is important to note that this constraining function might actually hinder analogical transfer performance (and thereby undermine the benefits of the constructing function). For example, Ross and Kilbane [28] showed that people tend to use the case to instantiate a generic representation. Consequently, this reliance on the case limits the referential flexibility of the generic representation to the single case presented. Nevertheless, proponents of the CASE&GENERIC-approach argue “that simultaneously using concrete and abstract (i.e., generic) problem representations during instruction promotes problem-solving success” ([24], p. 32).

### Which route to take? Prior research on transfer of knowledge about formal principles

Previous studies evaluating the transfer potential of the three approaches do not show a consistent pattern of results. For example, Kaminski and colleagues [11] reported an advantage of GENERIC over 2CASES_SIM, whereas Gick and Holyoak [19] found the opposite. Moreno, Ozogul, and Reisslein [24] found advantages for CASE&GENERIC over GENERIC for transfer performance, whereas Kaminski and colleagues ([13], Exp. 5) found no transfer advantage of CASE&GENERIC over GENERIC. The lack of a clear pattern in the literature cannot simply be traced back to differences in the domains studied (e.g., math vs science) or to superficial differences in the learning materials (e.g., different integration aids for different representations), but are rather subject to several substantial methodological differences across the studies.

First, studies differ in the assessment of learning and transfer. Some studies merely tested the ability to remember the contents of the learning materials by tasks directly adapted to the learning materials (e.g., [29]). If tasks are directly adapted to learning materials (e.g., by presenting tasks with the same representations as in the learning materials), participants are directly taught to the test [30]. Thus, the direct adaptation of an assessment does not reveal anything about the ability to transfer knowledge, but solely indicates how well learners are able to retrieve the contents of the learning material. Other studies limited transfer assessment to only one novel context (e.g., [11]). This limited assessment might entail the risk that this single context is more closely related to one of the learning materials (see [31], for a critical discussion as well as a replication and elaboration of the findings of Kaminski, et al. [11]). Furthermore, limited transfer assessment lacks external validity. Math teachers, for example, do not know which problems their students will encounter later on. Therefore, math teachers need to ensure that learners can flexibly use and apply math principles to a variety of contexts. Consequently, transfer performance should be assessed across a variety of tasks.

Second, studies differ in the timing of the assessment of transfer performance. Some studies measure transfer immediately after learning (e.g., [17]), others with a delay of several days (e.g., [12]). Immediate assessment lacks external validity, too. Moreover, it is possible that a particular approach fosters immediate learning and transfer, while it is detrimental for delayed transfer (for an overview see [32]). Teaching a novel formal principle in one lesson should (at least) result in a better task and transfer performance in the next lesson (e.g., a day or a week later). Thus, it is essential to measure transfer performance not only directly after learning, but also with a delay.

Third and most importantly, previous studies devoted to the different approaches have employed different control conditions. In studies investigating the GENERIC approach, transfer performance resulting from studying a generic formalism is often compared to performance resulting from studying a concrete instantiation (i.e., a single case) of the principle (e.g., [12]). In studies investigating the 2CASES_SIM approach, transfer performance is usually assessed by comparing the simultaneous with the sequential presentation of the same two cases (e.g., [29]). In studies investigating the CASE&GENERIC approach, mostly combinations of different representations are compared to single concrete or generic representation (e.g., [24]). These methodological differences make it impossible to reliably evaluate the strength and weakness of the different approaches by integrating previous research in a meta-analysis or a systematic review.

Taken together, there is a need to compare these three basic approaches to foster transfer of knowledge about formal principles within one experimental design. Such a comparison would not only help to evaluate the specific transfer potentials of the different approaches, but it would also help to render the underlying mechanism of transfer of relational knowledge about scientific principles more precisely. We therefore employed an experimental design which allows testing the impact of the three approaches on learning and immediate and delayed transfer performance with a tests comprised of various kinds of learning and transfer tasks in comparison to each other as well as against a common control condition.

## The Present Study

In the present study, we used the principles of propositional logic as learning domain to assess the transfer potential of the three approaches. Propositional logic comprises five basic principles. More specifically, propositional logic formalizes the truth functionality of the basic logic operators negation, conjunction, disjunction, conditional, and biconditional (see for example [33], for additional details). Understanding and being able to use and apply these principles is essential in various scientific domains (e.g., mathematics, electrical engineering, philosophy). This wide usage of propositional logic allows constructing a transfer test that contains several different kinds of tasks. In the following paragraphs, we will provide a first overview of the materials and tests used in the present research to illustrate and justify our research questions and hypotheses.

**Fig 1:**
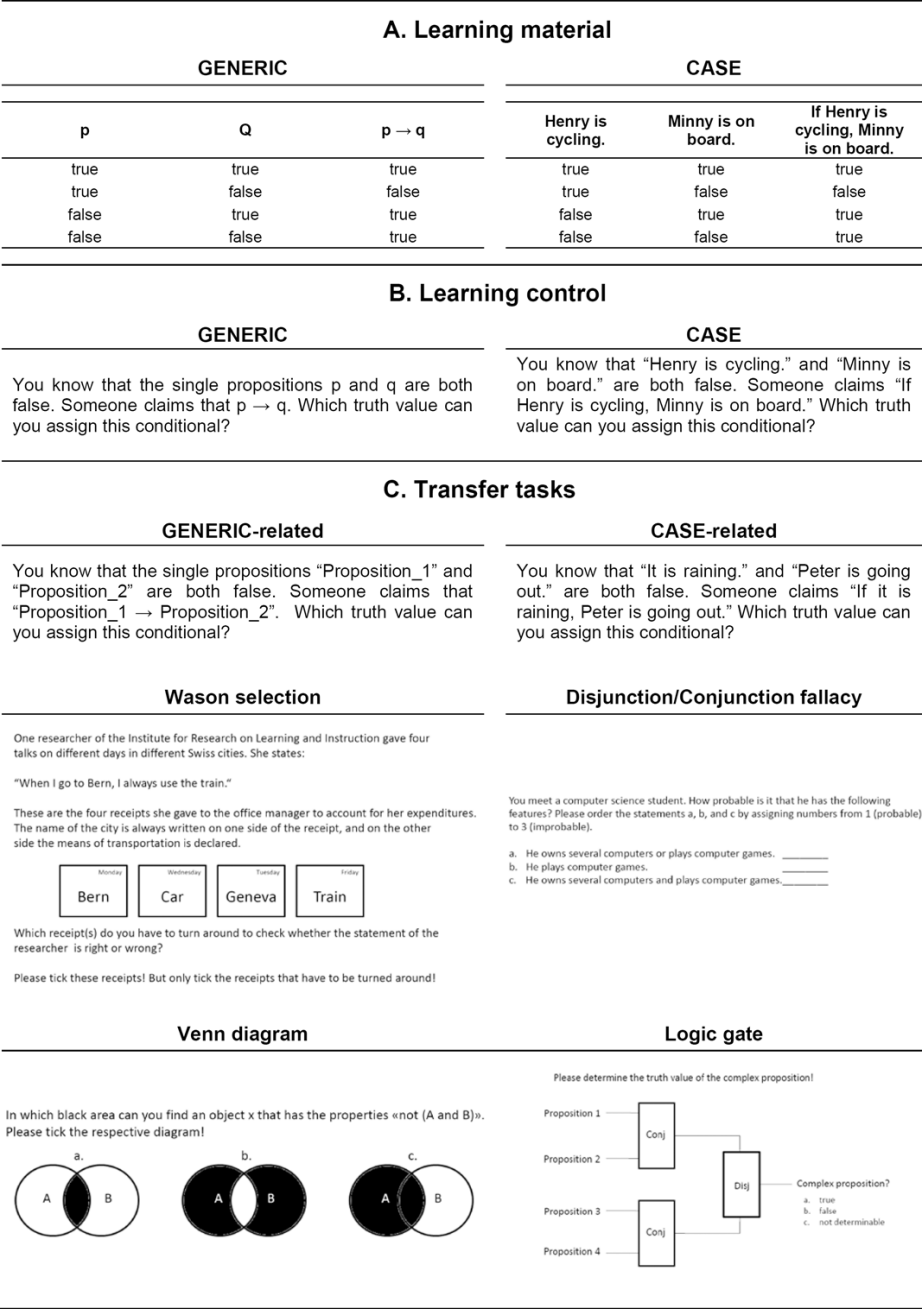
Examples of the learning materials, the learning control, and the different kinds of transfer tasks. Note: For the logic gates tasks, participants were given explanations for the abbreviations (e.g., conj = conjunction; disj = disjunction) as well as truth values for the basic propositions (i.e., Proposition 1, 2, 3, 4) to be able to answer the question.

We developed four different learning materials: GENERIC, 2CASES_SIM, CASE&GENERIC, and a control condition. The GENERIC learning materials represented the logic principles as generic formalisms using variables and symbols. For example, the conditional principle of propositional logic was represented as “p → q” in a truth table that fleshed out the truth functionality of this operator (Fig 1A’s left column shows exactly how this principle was represented in the GENERIC learning material). In the 2CASES_SIM materials, the variables and symbols used in the GENERIC learning material were simply instantiated by sentences (see the right column of Fig 1A for an example of a case) and two of these cases were presented simultaneously side by side (i.e., two truth tables with different sentences). Using simple sentences to illustrate logic principles is a conventional way to represent the principles in a concrete fashion, which is only a subtle variation to the variables and symbols used in the GENERIC learning materials. Using sentences to illustrate logic principles is a typical approach in logic textbooks (e.g., [33]) and this design of the cases also corresponds to many studies that investigated the effectiveness of 2CASES_SIM learning materials (e.g., [17, 19]). In the CASE&GENERIC learning material, principles were represented by a generic formalism and a single case simultaneously (exactly as displayed in Fig 1A). The learning material of the control condition (referred to as 2CASES_SEQ) represented the principles with the same two concrete cases used in the 2CASES_SIM learning materials. However, in the control condition the two cases were presented sequentially to prevent a direct comparison. This 2CASES_SEQ design corresponds to the control condition used in previous research on learning formal principles by comparing cases (see for example [17, 29]). This control condition provides a stronger test for the effectiveness of the different approaches as a single case control condition which is often used in studies that evaluated the GENERIC and the CASE&GENERIC approach. More importantly, presenting a single case inherently does not allow to abstract a generalizable schema as a learner cannot know which information presented in the case is relevant and which is not [9]. In a nutshell, learning materials differed only with regard to whether the principles of propositional logic were represented as symbols and variables and/or as sentences.

### The effectiveness of the learning materials

To assess whether the learning materials implementing the three approaches and the control condition successfully fostered learning and transfer of the principles, we compared the performance of participants who studied the GENERIC, 2CASES_SIM, CASE&GENERIC, and 2CASES_SEQ materials to the performance of participants who only answered the learning control or the transfer test without an intervention (NON-LEARNING conditions). As all approaches (including the control condition) have been shown to be beneficial for learning and transfer in previous research, the comparison to the NON-LEARNING conditions simply served as an implementation check. Of course, we expected that participants in all four learning conditions would outperform participants on learning and transfer who did not study any learning materials.

Furthermore, we tested participants’ initial understanding and learning of the principles to evaluate the quality of the learning material in all of the four conditions. To this aim, we applied two measures. First, we prompted participants to write self-explanations for each principle in all learning materials (e.g., [17, 34]). We coded the quality of these explanations to assess initial understanding and processing of the learning materials. Second, we measured the ability to remember the contents of the learning materials with a learning control test. The items of the learning control test were presented in the identical representational format that was used in the learning materials for each of the four groups (see Fig 1 and compare A and B for an example). That is, participants were directly taught to this test. Although the test versions differed in superficial features across the different conditions (i.e., whether principles were represented by symbols and variables or by sentences), the underlying structure of the tasks was identical across the different versions of the learning control test (compare the two example tasks in Fig 1B). We did not expect to find performance differences in initial understanding (i.e., in the quality of the self-explanations) and the ability to remember the principles of propositional logic (i.e., in the performance on the learning control test) across the different approaches (including the control condition). If we indeed find no differences in understanding and remembering the contents, this would indicate that the different learning materials are comparably intelligible initially. Potential differences in transfer performance could then in fact be traced back to the different learning processes triggered by the different representations used in the learning materials (and not to potential differences in the ease of processing, for example, that concrete materials might easier to process initially). This null hypothesis is derived from previous research comparing GENERIC and 2CASES_SIM (e.g., [11]) which indicated successful and comparable learning of the materials (but not transfer).

### Do the approaches differ with respect to learners’ transfer performance?

The ability to flexibly apply the knowledge encoded from the learning materials was assessed with a transfer test comprising various tasks. These different tasks situate the propositional logic principles in a variety of contexts. In contrast to the learning control test, the transfer test was identical (i.e., in structural and superficial features) for all conditions. This test was administered after participants answered the learning control test and was repeated one week later. This delayed transfer assessment served to check whether transfer performance differences would remain, decrease, or increase over time. The rationale for choosing a one-week-delay was that (at least in Europe) courses on the university level are scheduled on a weekly basis. Therefore, testing the content of the previous week is ecologically valid. The inconsistent picture of prior research did not allow deriving precise hypotheses regarding differences in transfer performance between GENERIC-, 2CASES_SIM-, and CASE&GENERIC-learners. However, we expected that the GENERIC-, 2CASES_SIM, and CASE&GENERIC-learners would outperform learners in the control condition (i.e., 2CASES_SEQ) and that this advantage would be stable over time.

### Assessing specific transfer effects to dissect underlying cognitive mechanisms

As we were primarily interested in evaluating the broad transfer potential of the different approaches across a wide range of tasks, we designed a subset of the transfer tasks to specifically test different theoretical predictions. Kaminski and colleagues ([11], p. 455) claimed that “(s)tudents might be better able to generalize mathematics concepts to various situations if the concepts have been introduced with the use of generic instantiations.” However, De Bock and colleagues [31] replicated Kaminski and colleagues’ study and their empirical results provided evidence that the transfer tasks used in Kaminski and colleagues study actually favored GENERIC learners (in their words “abstract learners”) because the transfer tasks were also situated in a generic context. De Bock and colleagues used exactly the same learning materials as Kaminski and colleagues, but extended the transfer test by additionally including transfer tasks that were situated in a concrete context. They indeed found that, while “abstract learners transferred what they had learned to a similar abstract context, this study (i.e., De Bock et al.’s study) shows also that students who learned from concrete examples transferred their knowledge into a similar concrete context.” (p. 109). Put differently, learning with concrete examples led to a better performance on concrete transfer tasks, while learning with abstract, generic materials led to a better performance on abstract transfer tasks. In the present study, we designed a subset of the transfer tasks with the aim to conceptually replicate De Bock and colleagues’ finding.

The subset of transfer tasks that were used to assess specific transfer effects were structurally identical to the learning control tasks, but differed in superficial characteristics. In particular, there were two kinds of transfer tasks that were specifically related to the learning materials: 10 GENERIC-related and 10 CASE-related tasks (see Fig 1C for examples of the two kinds, more detail on these tasks is provided in the method section). GENERIC-related transfer tasks substituted the generic instantiations used in the learning materials and the learning control test by different generic instantiations. CASE-related transfer tasks substituted the sentences used in the learning materials and the learning control test by novel sentences. Therefore, GENERIC-related tasks were more similar to the learning materials that contained generic instantiations (i.e., GENERIC and CASE&GENERIC) than to the learning materials based on cases only (i.e., 2CASES and BASELINE). In contrast, CASE-related tasks were more similar to the learning materials based on cases compared to the materials based on generic instantiations. Based on De Bock and colleagues’ [31] findings, we expected an interaction between learning material and task for GENERIC- and 2CASES-learners: GENERIC-learners should perform better on GENERIC-related tasks than on CASE-related tasks and 2CASES-learners should show the opposite pattern.

Moreover, the subset of transfer tasks additionally allows testing two conflicting theoretical predictions for the transfer performance of the CASE&GENERIC learners: different representations (as presented in the CASE&GENERIC learning materials) enable constructive schema abstraction or one representations constrains the other [28, 35]. On the one hand, the CASE&GENERIC-approach may result in the most successful performance on the GENERIC- and CASE-related transfer tasks, given that learners in this condition studied both kinds of representations in their learning materials and thus might construct and encode a more general schema [26]. If, on the other hand, the constraining function limits the referential flexibility of the generic representation to the single case (as suggested by [28]), then CASE&GENERIC-learners would show an inferior performance on GENERIC- and CASE-related transfer tasks in comparison to GENERIC- and 2CASES-learners.

## Method

### Ethics statement

All participants took part on a voluntary basis and gave written informed consent to their participation. The ethics committee of the institution where the study was conducted (ETH Zurich, Switzerland) approved the study. All data were collected and analyzed anonymously and can be found in the Supporting Information File (see S1 Dataset).

### Participants

We tested 150 native German-speaking university-undergraduates (61 males) majoring in a variety of subjects from a major city in Switzerland. Undergraduates studying mathematics, computer science, and philosophy were not allowed to participate as they are introduced to propositional logic early in their studies. Participants received financial compensation. They were randomly assigned to the four learning conditions (30 undergraduates per condition) and to the two NON-LEARNING conditions (with 20 undergraduates solving only the learning control test and 10 undergraduates solving only the transfer test). The numbers of participants in the NON-LEARNING conditions are lower, as these conditions merely served as an implementation check to evaluate whether participants in the learning conditions benefitted at all from studying the materials. Six participants of the learning conditions (each two in GENERIC, 2CASES_SIM, and 2CASES_SEQ) missed the delayed transfer test and were thus excluded from all statistical analyses.

At the end of the experiment, participants in all conditions were asked for biographic data and whether they had received any instruction on propositional logic prior to studying our learning materials. No participant reported prior instruction. Additionally, participants in the four learning conditions were tested on three subscales taken from a widely used German intelligence test (IST 2000R, [36]). The performance on these subscales (verbal intelligence: analogies; mathematical intelligence: arithmetic problems; figural-spatial intelligence: figure selections) served to assess whether randomization to conditions indeed resulted in comparable groups concerning general reasoning capabilities. No statistically significant differences between groups were detected on any of the three intelligence scales (analogies: *F*(3, 110) = 1.819, *p* = .148, *η*_*partial*_^*2*^ = .047; arithmetic problems: *F*(3, 110) = 1.196, *p* = .315, *η*_*partial*_^*2*^ = .032; figure selections: *F*(3, 110) = 0.269, *p* = .848, *η*_*partial*_^*2*^ = .007). Therefore, randomization created groups which are comparable with regard to basic cognitive abilities.

### Materials and procedure

The study comprised four phases: learning, learning control, immediate transfer, and delayed transfer. The first three phases were conducted within a first session. This first session took in total 3 hours including breaks. Participants were allowed to study the materials for a maximum of one hour. All participants managed to finish within this time limit. If a learner finished earlier, s/he was asked to go over the materials again, check their written explanations, and imagine preparing for an important exam in which s/he will be questioned about the contents of the learning materials. After the learning phase, a 15 minutes break followed. Subsequently, participants answered the learning control test for a maximum of 25 minutes. After working on the learning control test, another 15 minutes break followed. Afterwards, participants worked on the transfer test for the maximum of one hour.

One week later participants returned for the fourth phase, the delayed transfer assessment. Again, participants were allowed to work on the transfer test for the maximum of one hour. At the end of this second session, we also administered the intelligence scales and the biographic questionnaire.

#### Learning

Learning materials were presented as printed booklets. They comprised a short introduction to propositional logic which was identical for all conditions. The introduction was a short text describing why propositional logic is important for many domains to motivate learning. Importantly, this introduction did not mention or describe any of the to-be-learned principles.

On the following pages, truth tables represented the five basic principles of propositional logic (i.e., negation, conjunction, disjunction, conditional, and biconditional). The learning materials of the three experimental groups (GENERIC, 2CASES_SIM, CASE&GENERIC) and the control condition (2CASES_SEQ) differed only in the representations used for the principles in the truth tables. That is, the truth tables of the GENERIC-material represented the principles with variables for propositions and symbols for the operators (see Fig 1A GENERIC). In contrast, the variables and symbols were instantiated with simple sentences in the 2CASES_SIM- and 2CASES_SEQ-materials (see Fig 1A CASE). In the 2CASES_SIM-material, the two cases were presented simultaneously (i.e., two truth tables side by side on a single page). For the second case, different sentences were used for instantiating the same principle as in the first case. In the 2CASES_SEQ-material, the cases were presented sequentially. That is, participants actually worked with two booklets. In the first booklet, truth tables presented the first case; in the second booklet, truth tables presented the second case. Importantly, participants had to return the first booklet before receiving the second booklet to prevent a direct comparison of cases. In the CASE&GENERIC-material, one case was presented simultaneously with the GENERIC-representation (i.e., side by side on a single page, exactly as it is represented in Fig 1A).

In all learning materials, participants were prompted to write explanations for each principle to foster constructive engagement with the contents (e.g., [37, 38]). The prompts differed slightly between learning conditions to align with the different representations used in the materials. In the 2CASES_SIM condition, the prompt was: “Look at the first and the second example. Which similarities do you notice? Describe them briefly!” In the CASE&GENERIC condition, the prompt was: “Look at the example with sentences and at the example with variables. Which similarities do you notice? Describe them briefly!” In the GENERIC and the 2CASES_SEQ condition, prompts were identical: “Look at the example. What stands out? Describe your observation briefly!”

#### Learning Control

The learning control test comprised 24 multiple-choice tasks. Every tasks required participants to judge the validity of a problem based on the principles of propositional logic by choosing one out of three answer options: *logically valid*, *logically invalid*, and *logically undetermined*.

The tasks of the learning control test differed in superficial characteristics between conditions; but, the structure of the tasks was identical across conditions. That is, learners who studied the principles of propositional logic by means of cases (2CASES_SIM and 2CASES_SEQ) had to solve tasks constructed with the same sentences that had been used for the cases in the learning materials. Learners who studied the principles of propositional logic by means of a generic formalism (GENERIC) had to solve tasks which used the same variables and symbols that represented the principles in the learning materials. For learners who studied both a case and a generic formalism (CASE&GENERIC), half of the learning control test tasks were based on sentences and the other half was based on variables. As these tasks were thus directly adapted to the representations used in the learning materials, answering these tasks basically required recalling the truth tables studied (for examples of the tasks see Fig 1B and compare GENERIC and CASE).

#### Transfer

In contrast to the learning control test, the transfer test was identical for all conditions. The test comprised 44 transfer tasks. Of these tasks, 20 tasks were designed to specifically assess specific transfer effects of the 2CASES_SIM, GENERIC, and the CASE&GENERIC approach. This subset of transfer tasks comprised tasks that are related in superficial characteristics to the different representations used in the learning materials. The remaining 24 items of the transfer test represented the principles of propositional logic in novel contexts that were not introduced before and were unrelated to the representations used in the learning materials. All transfer tasks were presented intermixed in a printed booklet.

The transfer tasks that were related to representations used in the learning materials were structurally identical to 10 tasks used in the learning control test but superficial details were changed. These ten tasks were presented in two versions each. GENERIC-related transfer tasks substituted the variables used to represent propositions in the GENERIC learning materials and the learning control test (i.e., “p” and “q”) by other variables (i.e., “Proposition 1” and “Proposition 2”, see Fig 1 and compare GENERIC A, B, and C). CASE-related transfer tasks substituted the sentences used to represent the principles in the CASE-based learning materials and the learning control test by different sentences (see Fig 1 and compare CASE A, B, and C). These two kinds of transfer tasks were presented in multiple choice format with three answer options to indicate the logical validity of the scenario described in the task like in the learning control test tasks (i.e., *logically valid*, *logically invalid*, and *logically undetermined*).

The transfer tasks that are not related to representation used in the learning materials comprised four different kinds of tasks: Wason selection tasks, disjunction/conjunction fallacy tasks, Venn diagram tasks, and logic gate tasks (see Fig 1C for examples). Venn diagram and logic gates tasks were both presented as multiple-choice tasks with three answer options each. The transfer test contained eight Venn diagram and eight logic gate tasks. Venn diagram tasks required participants to transfer their knowledge about the principles of propositional logic to representations used in set theory. The Venn diagram tasks always provided three diagrams with different areas blackened. Participants had to decide which diagram represented the logical relations specified in the task. Logic gates tasks related propositional logic to representations used in computer science and engineering. A logic gate represents a logical operator which combines one or more inputs to produce a single output. Note that the representations used to symbolize logic gates in these tasks did not exactly resemble the ones actually used in computer science and engineering where specific representations are used for logical principles [39]. These specific representations were simplified to rectangles in which an abbreviation indicated the logic. This simplification was necessary to circumvent having to teach new representations within the transfer test. For logic gates tasks, participants were asked to determine the truth value of a complex proposition (as either *true*, *false*, or *not determinable*) that resulted from the connection of simple propositions with different operators.

Wason selection tasks (e.g., [40]) and conjunction/disjunction fallacy tasks (e.g., [41]) have been widely used to demonstrate that human thinking often does not adhere to the formal rules of logic. Thus, these tasks are difficult but excellent for assessing the ability to transfer the principles of propositional logic. The test included four Wason’s selection tasks and four conjunction/disjunction fallacy tasks. In all Wason’s selection tasks, participants saw four cards printed in the booklet of the transfer test. The cards were described to have different information on the front and the back. Participants were instructed to tick the cards that had to be turned around to determine whether a conditional rule was true or false. All Wason’s selection tasks required to tick two cards for a correct answer (in logic talk, these two cards represent the modus ponendo ponens and the modus tollendo Tollens, see for example [42]). A task was scored as correct, only if both cards had been ticked.

Also, four versions of the conjunction/disjunction fallacy task were used (the construction of the these tasks was inspired by [43]). These tasks always started with a short cover story. Afterwards, participants read three statements about a subject mentioned in the cover story and had to order these statements by their probability. One of the statements described a single property of the object, another statement described the conjunction of this property with a second property, and the last statement described the disjunction of these two properties. From a logical point of view, the conjunction is least probable, the disjunction is most probable. The task was scored as correct only if participants provided the correct order over all three statements.

#### NON-LEARNING conditions

To assess whether studying the learning materials resulted in any learning and transfer gains, we additionally gave either only the learning control or the transfer test to participants who did not study any learning material. The time to solve the tests in the NON-LEARNING conditions was the same as for participants in the learning conditions (i.e., max. 25 minutes for the learning control test and max. 60 minutes for the transfer test).

## Results

For all analyses, we predefined *p* < .05 to indicate statistical significance. Specific comparisons of GENERIC, 2CASES_SIM, and CASE&GENERIC to either NON-LEARNING or the control condition (2CASES_SEQ) are based on clear unidirectional hypotheses derived from the prior research literature regarding the transfer performance, namely that NON-LEARNING and 2CASES_SEQ should be outperformed. Therefore, we computed one-tailed significance tests when comparing the performance of GENERIC, 2CASES_SIM, and CASE&GENERIC to 2CASES_SEQ, or NON-LEARNING, respectively. All other comparisons (i.e., between GENERIC, 2CASES_SIM, and CASE&GENERIC) are not based on directional hypotheses and therefore, we computed two-tailed significance tests for these comparisons. Pairwise comparisons are presented with Cohen’s *d* as effect size measure. When using multifactorial analyses, *η*_*partial*_^*2*^ is provided as effect size measure for the omnibus effect of a factor within the multifactorial model.

To check whether the learning materials support learning and transfer of propositional logic at all, we first compared the performance of the learning conditions (GENERIC, 2CASES, CASE&GENERIC, and 2CASES_SEQ) for the learning control and the transfer test to the performance of the NON-LEARNING participants (see Table 1 for means and standard deviations). Studying the learning materials led to strong advantages compared to NON-LEARNING in the learning control test performance (GENERIC: *t*(46) = 4.895, *p* < .001, *d* = 1.44; 2CASES_SIM: *t*(46) = 4.692, *p* < .001, *d* = 1.39; CASE&GENERIC: *t*(48) = 3.136, *p* = .002, *d* = 0.91; 2CASES_SEQ: *t*(46) = 5.097, *p* < .001, *d* = 1.50) and the transfer test performance (GENERIC: *t*(36) = 3.732, *p* = .001, *d* = 1.24; 2CASES_SIM: *t*(36) = 3.868, *p* < .001, *d* = 1.29; CASE&GENERIC: *t*(38) = 2.687, *p* = .006, *d* = 0.87; 2CASES_SEQ: *t*(36) = 3.028, *p* = .003, *d* = 1.01). Thus, all learning materials supported learning and transfer of the principles of propositional logic in comparison to not studying any learning material. Of course, the effect that participants who studied one of the learning materials did actually learn is rather trivial. Nevertheless, these advantages reinstate previous research claiming that the approaches do indeed foster the ability to transfer knowledge about formal principles and thus indicate that we successfully implemented the different approaches in the learning materials.

**Table 1:** Mean sum score for quality of explanations and mean percentages of correct answers (standard deviations) for the learning control test and the immediate and delayed transfer test.

		GENERIC (*N* = 28)	2CASES_SIM (*N* = 28)	CASE& GENERIC (*N* = 30)	2CASES_SEQ (*N* = 28)	NON-LEARNING^a^
**Explanations (max. 10)**		4.4 (3.5)	3.8 (3.4)	4.3 (3.6)	4.3 (3.2)	---
**Learning control**		73.6 (12.0)	72.6 (11.6)	68.1 (12.8)	72.8 (10.6)	57.0 (10.5)
**Transfer**	**immediate**	58.5 (12.8)	62.2 (15.5)	54.3 (13.4)	55.2 (12.7)	42.0 (8.8)
	**delayed**	61.2 (14.9)	64.6 (16.1)	53.8 (14.0)	57.9 (12.8)	---

^**a**^ There were two different NON-LEARNING groups: One group answered only the learning control test (*N* = 20); the other group answered only the transfer test once (*N* = 10).

### The effectiveness of the learning materials

Quality of explanations and performance in learning control test served to check whether the different learning materials were comparably intelligible. All explanations were coded into three categories by two independent coders: (1) correct explanation of the underlying principle, (2) explanations that only reproduced the information given in the learning materials, and (3) wrong, unreadable, or missing explanations and were scored with 2, 1, and 0 points, respectively (i.e., max. 10 points as participants had to explain the five principles of propositional logic). The inter-coder reliability was satisfactory (Cohen’s *Kappa* = .83). Disagreements were solved through discussion. The quality did not substantially differ between the four groups (*F*(3, 110) = .607, *p* = .612; see Table 1 for means). For the learning control test, the mean solution rates (see Table 1) did also not substantially differ between the four learning groups (*F*(3, 110) = 1.991, *p* = .142). These results indicate that all learning programs were comparably intelligible and that the participants in the different learning conditions were able to remember the contents of the learning materials to a comparable amount as expected.

### Do the approaches differ with respect to learners’ transfer performance?

The comparable performance in the learning control test allowed us to use the performance in the learning control test as a covariate for the analyses of the transfer test. Using the initial learning performance as a covariate increases the power of the transfer performance analyses. Furthermore, models including this covariate provides a more pure assessment of the transfer potential of the different approaches by reducing “noise” caused by interindividual differences in response to the learning materials themselves [44]. We z-standardized the performance in the learning control test within conditions and used this standardized variable as a covariate in all analyses of the transfer test. Note that the pattern of statistically significant results for the transfer test analyses remains similar if computed without z-standardizing the covariate. The covariate did not interact with the learning conditions in any of the following analyses (all *p*s > .05); that is, regression slopes can be assumed to be homogeneous. Furthermore, the distribution of transfer scores within conditions did not deviate significantly from normal distributions (all *p*s > .05). Consequently, successful learning is linearly related to transfer performance across time in all conditions.

In all following transfer performance analyses, the repeated assessment of the transfer test was treated as a within-subject factor. If this factor turned out to be statistically significant or interacted with one of the other factors, we computed separate analyses for the immediate and delayed transfer assessment to scrutinize transfer performance changes over time.

**Fig 2:**
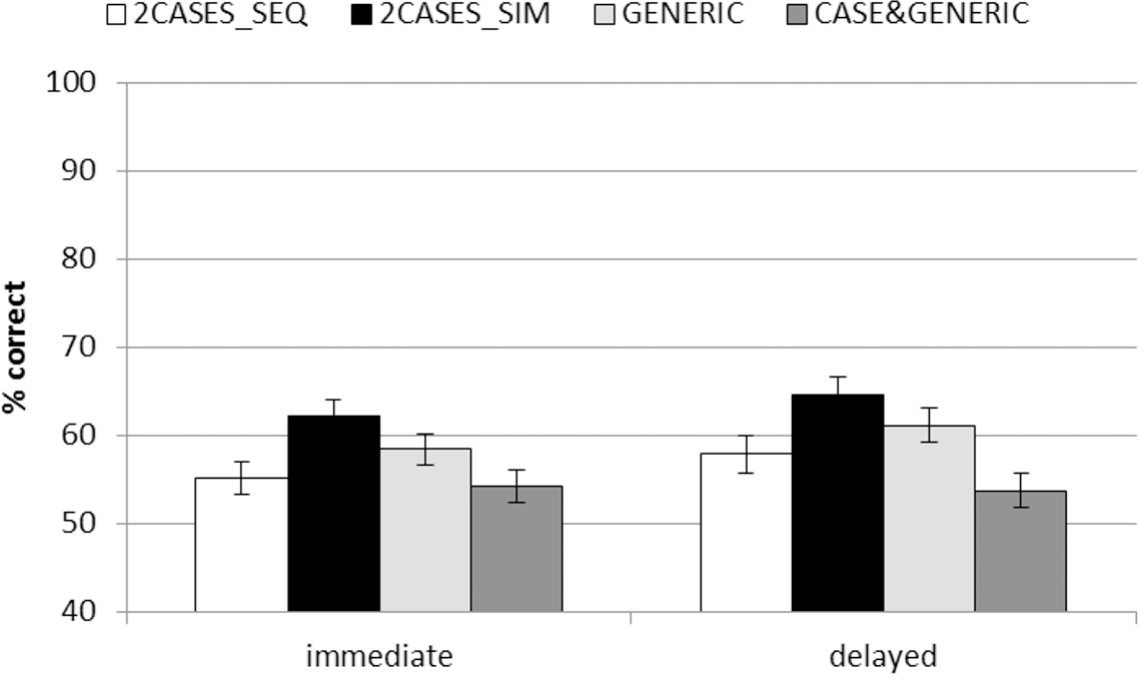
Marginal estimated means for immediate and delayed transfer performance for all learning conditions. Error bars indicate standard error of the mean.

We analyzed transfer performance across all 44 transfer tasks (see Fig 2 for a graph representing the marginal estimated means) with a 2 (time: immediate transfer and delayed transfer) x 4 (learning conditions) ANCOVA (with the covariate: learning control test performance). This analysis revealed statistically significant effects of the covariate (*F*(1, 109) = 99.505, *p* < .001, *η*_*partial*_^*2*^ = .477), time (*F*(1, 109) = 6.031, *p* = .016, *η*_*partial*_^*2*^ = .052), and condition (*F*(3, 109) = 4.922, *p* < .001, *η*_*partial*_^*2*^ = .477). All other effects and interactions were not significant. The significant effect of the covariate indicates that the performance in the learning control test strongly predicted performance in the transfer test. The significant effect of time indicates a very small improvement of performance over time across all conditions (*p* = .016, *d* = 0.13).

Due to this change over time, we computed two specific ANCOVAs to compare the performance of the different conditions for immediate and delayed transfer separately. Both analyses showed a significant influence of the covariate (immediate: *F*(1, 109) = 96.073, *p* < .001, *η*_*partial*_^*2*^ = .468; delayed: *F*(1, 109) = 76.896, *p* < .001, *η*_*partial*_^*2*^ = .414) and significant effects of conditions (immediate: *F*(3, 109) = 3.642, *p* = .015, *η*_*partial*_^*2*^ = .091; delayed: *F*(3, 109) = 4.993, *p* = .003, *η*_*partial*_^*2*^ = .121). Pairwise comparisons for the immediate transfer performance comparing the different learning conditions to the control condition showed that only 2CASES_SIM outperformed 2CASES_SEQ (*p* = .011, *d* = .49), while GENERIC (*p* = .229, *d* = .26) and CASE&GENERIC (*p* = .721, *d* = -.07) failed to outperform 2CASES_SEQ. Furthermore, CASE&GENERIC was outperformed by 2CASES_SIM (*p* = .003, *d* = .54).

The pairwise comparisons for the delayed performance indicated that the differences found for immediate transfer performance were stable. That is, again only 2CASES_SIM outperformed the 2CASES_SEQ (*p* = .027, *d* = .46). GENERIC (*p* = .266, *d* = .24) and CASE&GENERIC (*p* = .160, *d* = -.30) still failed to outperform the control condition. Also, the advantage of 2CASES_SIM over CASE&GENERIC remained stable (*p* < .001, *d* = .72). In addition, GENERIC also outperformed CASE&GENERIC in the delayed transfer assessment (*p* = .012, *d* = .51).

Taken together, the results for transfer performance across all transfer tasks showed unexpectedly that GENERIC and CASE&GENERIC failed to perform better than the control condition (2CASES_SEQ); an advantage over this control condition was only detected for the 2CASES_SIM learners. CASE&GENERIC performed inferior to 2CASES_SIM on both immediate and delayed transfer, and was also outperformed by GENERIC in the delayed transfer. Furthermore, while the overall ANCOVA indicated a slightly increasing performance over time, inspection of the solutions rates indicates that only the performance of 2CASES_SIM, GENERIC, and 2CASES_SEQ learners increased, but that CASE&GENERIC learners actually slightly decreased in their transfer performance.

### Analyzing specific transfer effects

**Fig 3:**
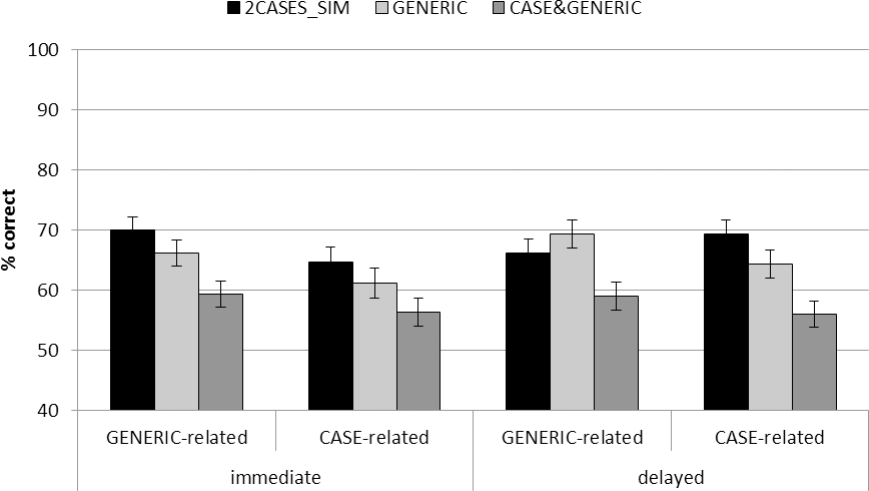
Marginal estimated means for immediate and delayed performance on CASE-related and GENERIC-related transfer tasks. Error bars indicate standard error of the mean.

To assess specific transfer effects of the three experimental condition (2CASES_SIM, GENERIC, and CASE&GENERIC), we additionally analyzed the transfer performance on a subset of the transfer tasks (i.e., CASE-related and GENERIC-related transfer tasks). We first computed a 2 (time: immediate transfer and delayed transfer) x 2 (task: CASE-related and GENERIC-related) x 3 (learning conditions: 2CASES_SIM, GENERIC, and CASE&GENERIC) ANCOVA (with the covariate: learning control performance) treating time and task as repeated measure factors (see Fig 3 for a graph representing the marginal estimated means). This analysis shows statistically significant effects of the covariate (*F*(1, 82) = 127.910, *p* < .001, *η*_*partial*_^*2*^ = .609), task (*F*(1, 82) = 7.899, *p* = .006, *η*_*partial*_^*2*^ = .088), and condition (*F*(2, 82) = 9.659, *p* < .001, *η*_*partial*_^*2*^ = .191). No other effects or interactions were detected. Thus, we did not conduct specific analyses for the two times of assessment. The significant effect of the covariate indicates that the performance in the learning control test was highly predictive for the performance on the transfer tasks which were related to the learning materials. The effect of task indicates that CASE-related transfer tasks (solution rate: 61.9%) were slightly more difficult than GENERIC-related transfer tasks (65.0%; *p* = .006, *d* = 0.19). Pairwise comparisons for the effect of condition collapsing across both kinds of tasks revealed that CASE&GENERIC (solution rate: 57.7%) was outperformed by 2CASES_SIM (solution rate: 67.5%; *p* < .001, *d* = 0.71) and GENERIC (solution rate: 65.2%; *p* = .002, *d* = 0.53). This result indicates that the simultaneous presentation of the case and the generic representation in the CASE&GENERIC learning material did not support the construction of a more general schema as well as 2CASES_SIM, but that the case rather constrained the referential flexibility of the generic representation (as indicated by the superior performance of the GENERIC learners).

To test for the hypothesized interaction between task (CASE-related vs. GENERIC-related) and learning condition (2CASES_SIM vs. GENERIC), we additionally computed an analysis including only 2CASES_SIM and GENERIC. The 2 (time: immediate transfer and delayed transfer) x 2 (task: GENERIC-related, CASE-related) x 2 (learning condition: 2CASES_SIM, GENERIC) ANCOVA (with the covariate: learning control performance) showed a main effect of the covariate as in the previous analyses (*F*(1, 53) = 112.101, *p* < .001, *η*_*partial*_^*2*^ = .679) and an effect of task (*F*(1, 53) = 4.977, *p* = .030, *η*_*partial*_^*2*^ = .086). Furthermore, two interactions, although not statistically significant, seemed to qualify the effects of task and time (time x task: *F*(1, 53) = 3.287, *p* = .075, *η*_*partial*_^*2*^ = .058; time x task x condition: *F*(1, 53) = 3.287, *p* = .075, *η*_*partial*_^*2*^ = .058).

To explore these interactions, we computed two 2 (task) x 2 (learning condition) ANCOVAs, one for each point of assessment. The analysis for the immediate transfer performance only revealed main effects of the covariate (*F*(1, 53) = 106.232, *p* < .001, *η*_*partial*_^*2*^ = .667) and task (*F*(1, 53) = 6.714, *p* = .012, *η*_*partial*_^*2*^ = .112). The effect of task indicates that CASE-related transfer tasks were slightly more difficult than GENERIC-related transfer tasks (solution rates: 62.9% vs. 68.0%; *p* = .012, *d* = 0.29). In contrast, the analysis for the delayed transfer performance revealed the expected task x condition interaction (*F*(1, 53) = 6.740, *p* = .012, *η*_*partial*_^*2*^ = .113) and a significant effect of the covariate (*F*(1, 53) = 62.167, *p* < .001, *η*_*partial*_^*2*^ = .113). The interaction for the delayed transfer performance indicates that 2CASES_SIM-learners performed better on CASE-related transfer tasks while GENERIC-learners performed better on GENERIC-related transfer tasks (see Fig 3).

## Discussion

We evaluated three different approaches (GENERIC, 2CASES_SIM, and CASE&GENERIC) for designing learning materials to foster transfer of knowledge about formal principles important for scientific reasoning. To this aim, participants studied the principles of propositional logic (i.e., the truth functionality of the basic logic operators negation, conjunction, disjunction, conditional, and biconditional). In the GENERIC learning material, principles were presented as generic formalisms only. In the 2CASES_SIM learning material, the variables and symbols of the generic formalisms were instantiated with simple sentences and participants processed two cases (i.e., two different combinations of simple sentences) simultaneously to allow direct comparison of the two cases. Finally, in the CASE&GENERIC learning material, a principle was presented by a combination of a generic formalism (like in the GENERIC learning material) and a single case (one of the cases used in the 2CASES_SIM learning material). We compared the learning and transfer performance of GENERIC, 2CASES_SIM, and CASE&GENERIC-learners to each other, to a control condition (2CASES_SEQ), and to learners who did not study any learning materials (NON-LEARNING).

As expected, all learning materials (including the control condition) resulted in significant learning and transfer compared to participants who did not study any learning material (NON-LEARNING). These results indicate that all learning materials supported students in learning about propositional logic and in being able to generalize this knowledge to some extent. Moreover, we did not find differences between GENERIC, 2CASES_SIM, CASE&GENERIC, and 2CASES_SEQ-learners with regard to understanding (i.e., the quality of explanations) and remembering the contents (i.e., performance on the learning control test) of the learning materials indicating that all materials were comparably intelligible.

We did however find differences in the ability to transfer knowledge gained from studying the learning materials. It is important to note that 2CASES_SIM-learners did not directly outperform GENERIC-learners. Nevertheless, 2CASES_SIM was the only approach which resulted in a reliable advantage in the performance across all transfer tasks over time compared to the control condition (2CASES_SEQ). Furthermore, it turned out that the CASE&GENERIC-approach was the least successful approach tested: Participants who learned according to this approach did not only fail to outperform the control condition, but they were also outperformed by participants in the GENERIC (on the delayed transfer assessment) and 2CASES_SIM (on the immediate and the delayed assessment) conditions.

Here, it is important to recall that the learners in the control condition (2CASES_SEQ) processed the same two cases as the 2CASES_SIM learners, but the cases were presented sequentially. That is, the amount of information presented in the 2CASES_SEQ is equal to 2CASES_SIM and comparable to CASE&GENERIC in which also two representations of a principle are presented. This control condition is stronger than the control conditions in most previous studies investigating the GENERIC- and CASE&GENERIC-approaches which often employed single concrete case control conditions. We like to emphasize here again that a single concrete case condition does not allow participants to abstract a more general schema which could enable transfer because learners cannot know from processing a single case which properties of the case are relevant and which properties are irrelevant aspects of an underlying principle [9]. Thus, a single case control condition is, in general, problematic for research on transfer.

With our study, we contribute to the large body of research on the 2CASES_SIM approach by showing (1) that the simultaneous comparison of cases indeed leads to a successful transfer performance across a wide range of transfer tasks which is (2) stable over time and that (3) the 2CASES_SIM-approach was the only approach in our study that managed to outperform the control condition. Indeed, previous research has shown that the simultaneous presentation of two cases enhances transfer performance in comparison to the sequential presentation (see [20], for a meta-analysis). If learners are prompted to directly compare the cases, they engage in a structural alignment process which highlights similarities and differences between the cases and allows the abstraction of a relational schema (e.g., [6]). Abstraction of a relational schema requires comparing cases that share (if possible) only the relational structure of the to-be-learned principle while the concrete instantiation of the relational structure (e.g., the specific sentences used to instantiate a logical conjunction or specific objects used to illustrate Newton’s second law) or the context in which the cases are embedded should be different. Designing cases in such a way would help learners to construct a schema that only contains the shared relational structure of the cases, but no superficial variations of the cases that are irrelevant for the understanding, use, and application of the principle.

The CASE&GENERIC approach did not support the abstraction of a generalizable schema as well as the 2CASES_SIM approach. Consistent with previous research our results indicate that integrating different representation as demanded in the CASE&GENERIC material is difficult [26] and might therefore require stronger integration aids than the explanation prompts we provided in the learning materials (which were, however, sufficient to support schema abstraction in 2CASES_SIM learners). Recall that the CASE&GENERIC learning material contained all the information available in the GENERIC learning materials plus an additional case. Despite this additional information, CASE&GENERIC-learners transferred their knowledge worse than GENERIC learners, which became especially evident in the analyses of the subset of transfer tasks that were closely related to the learning materials. Here, our findings are consistent with the hypothesis that different representations can also constrain one another’s interpretation [27]. Consequently, CASE&GENERIC-learners seem to have only matched the concrete, idiosyncratic features of the case to the symbols and variables of the generic representation without abstracting or elaborating a general schema. In contrast, GENERIC-materials might have engaged in constructive elaboration of learning materials with prior knowledge (e.g., triggering the question “What might this symbol stand for?”) while the case presented in the CASE&GENERIC-materials made this elaboration process unnecessary. Even worse, the case seemed to limit the referential flexibility of the generic instantiation (indicated by the superior performance of GENERIC learners). Moreover, our results reinstate findings by Ross and Kilbane [28] who showed that learners strongly relied on a case when trying to solve a transfer problem if the case was presented together with a generic representation during the learning phase. We hypothesize that this constraining function is especially pronounced if single different representations for one principle are provided (e.g., [24, 25]). Single different representations of a concept or principle (like a case, a graph, or a formula) might engage learners in matching the representations superficially, but might not prompt learners to abstract from or elaborate materials even when supported by explanation prompts.

The analysis of the subset of transfer tasks that were related to the representations used in the learning materials further allowed us to conceptually replicate previous studies [11, 31]. GENERIC-learners performed better on GENERIC-related than on CASE-related tasks while 2CASES_SIM-learners showed the opposite pattern (see [31], for exactly the same pattern of results), even though this pattern of results was only found to be statistically significant in the delayed transfer performance. This interaction indicates that both learning materials resulted in the encoding of knowledge representations that preserved specific properties of the representations used in the learning materials. That is, learning a principle from a generic representation seems to lead to the encoding of a knowledge representation which preserves the features that the principle was represented with symbols and variables (also see [16]). Learning a principle by comparing two cases based on sentences seems to lead to the encoding of a knowledge representation which preserves the feature that both cases were based on sentences. Consequently and that is what our results indicate, transfer is facilitated when transfer tasks share these similarities to the representation used in the learning materials (see also [45], who elegantly identified different transfer mechanisms by varying the similarity between learning and transfer tasks).

Generic representations in learning materials thus might not be as powerful as some researchers suggested (e.g., [11, 15]). Importantly however, we do not claim that a generic representation of a principle should not be included in learning materials. GENERIC learners were able to generalize their knowledge and knowing and using generic representation becomes extremely important or even indispensable for solving more complex problems (e.g., by allowing the application of computational procedures). Future research is thus needed to examine how a generic representation can be integrated in learning materials. For example, one could simultaneously present several cases along with the generic representations or one could present case comparisons and generic representations sequentially (e.g., [46–48]).

The present results highlight the importance of assessing transfer by multiple tasks and by comparing different approaches in one study. However, several points are left unresolved and need further scrutiny. First, our results indicate that performance differences between conditions became slightly larger over time. For example, the interaction in performance for 2CASES_SIM and GENERIC on the two kinds of transfer tasks related to the learning materials was only found in the delayed test. It is unclear why this is the case. One possible explanation might be that memory retrieval one week later is more strongly influenced by superficial features of transfer tasks than directly after learning. However, the design of the present study does not allow evaluating this assumption. Future studies are needed that competitively test the different approaches and vary the temporal distance between learning and testing without repeating the transfer test (e.g., assessing transfer one day, two days, or a week after learning). In any case, we like to reinstate that it is indispensable to measure transfer with a delay (see also [32]). Relying on a transfer assessment immediately after learning lacks external validity, that is, it simply does not generalize to real educational settings that pursue the goal of teaching principles and concepts.

Second, it is unclear to what extent participants’ transfer performance benefitted from working on the learning control test. It has been shown that testing can enhance transfer (e.g., [49]) and our findings indicate that the learning control test performance which was used as a covariate strongly predicted transfer performance. Therefore, it is possible that testing was less effective in the CASE&GENERIC condition, because this condition involved more variability in the learning control test. This higher degree of variability may have increased cognitive load and may have made it harder to benefit from testing in the CASE&GENERIC condition in comparison to the other learning conditions. However, the learning control test performance (i.e., the covariate) did not interact with the different learning conditions. Thus, the testing effect seems to play a similar role in all conditions. We applied a learning control test and a transfer test to demonstrate that the mere assessment of learning and retention does not help to discriminate between the different approaches. However, future studies should evaluate whether and to what extent solving the learning control test (as well as the repeated assessment of the transfer test as discussed above) contributed to the transfer performance.

Third, it is possible that the 2CASES_SEQ-learners also engaged in comparisons when processing the two cases sequentially. That is, they could have compared their knowledge representation encoded from processing the first case with the representation encoded from processing the second case. Such an internal comparison process has been suggested by memory and category learning studies investigating spacing (e.g., [50]) and interleaving effects (e.g., [51]). Indeed, implicit comparisons cannot be excluded and this implicit comparison might serve as an explanation for the decent transfer performance of the 2CASES_SEQ-learners. However, the sequential presentation of examples is the standard control condition in research devoted to the 2CASES_SIM-approach (e.g., [17, 52]). This research has consistently revealed advantages in abstracting a common scheme from two cases if they are presented simultaneously as this allows direct structural alignment (also see the meta-analysis by [20]). In other words, comparison processes may be prompted more directly by presenting cases in parallel rather than sequentially. At the same time, it is important to note that most of the memory and category learning research is difficult to compare to the present research as these studies usually investigate learning of feature-based categories over multiple trials ([4], for an extended critique). In the present research the focus was on learning relational formal principles with an exposure to only one (i.e., in the GENERIC approach) or two instantiations of a principle (i.e., in the 2CASES_SIM, CASE&GENERIC, and 2CASES_SEQ approaches). Future research is needed to clearly identify commonalities and differences in learning feature-based and relational concepts or categories (see [53], for a promising step in this direction).

To our knowledge the present study is the first to directly test the three most basic approaches to foster transfer of formal principles against each other by using a broad range of transfer tasks. The results have implication for educational settings which aim at designing learning materials for teaching generalizable, formal principles. These kinds of principles are central to science and mathematics education. The ability to transfer these kinds of principles requires learners to engage with learning materials that support schema abstraction (i.e., encoding of flexible knowledge representations). The results indicate that a promising way to introduce relational principles like the principles of propositional logic is to engage learners in the direct comparison of cases instantiating these principles.

## Supporting Information

S1 Dataset The dataset contains all data of the present study. (SAV)
